# PNPLA3 I148M associations with liver carcinogenesis in Japanese chronic hepatitis C patients

**DOI:** 10.1186/s40064-015-0870-5

**Published:** 2015-02-13

**Authors:** Kazunori Nakaoka, Senju Hashimoto, Naoto Kawabe, Yoshifumi Nitta, Michihito Murao, Takuji Nakano, Hiroaki Shimazaki, Toshiki Kan, Yuka Takagawa, Masashi Ohki, Takamitsu Kurashita, Tomoki Takamura, Toru Nishikawa, Naohiro Ichino, Keisuke Osakabe, Kentaro Yoshioka

**Affiliations:** Department of Liver, Biliary Tract and Pancreas Diseases, Fujita Health University, 1-98 Dengakugakubo, Kutsukakecho, Toyoake, Aichi 470-1192 Japan; Faculty of Medical Technology, School of Health Sciences, Fujita Health University, Toyoake, Aichi 470-1192 Japan

**Keywords:** PNPLA3, HCC, Chronic hepatitis C, SNP, Cirrhosis, HCV

## Abstract

**Aim:**

To investigate associations between patatin-like phospholipase domain-containing 3 (PNPLA3) genotypes and fibrosis and hepatocarcinogenesis in Japanese chronic hepatitis C (CHC) patients.

**Methods:**

Two hundred and thirty-one patients with CHC were examined for PNPLA3 genotypes, liver stiffness measurements (LSM), and hepatocellular carcinoma (HCC) from May 2010 to October 2012 at Fujita Health University Hospital. The rs738409 single nucleotide polymorphism (SNP) encoding for a functional PNPLA3 I148M protein variant was genotyped using a TaqMan predesigned SNP genotyping assay. LSM was determined as the velocity of a shear wave (Vs) with an acoustic radiation force impulse. Vs cut-off values for cirrhosis were set at 1.55 m/s. We excluded CHC patients with a sustained virological response or relapse after interferon treatment.

**Results:**

PNPLA3 genotypes were CC, CG, and GG for 118, 72, and 41 patients, respectively. Multivariable logistic regression analysis selected older age (OR = 1.06; 95% CI: 1.03–1.09; p < 0.0001), higher body mass index (BMI) (OR= 1.12; 95% CI: 1.03–1.22; p = 0.0082), and PNPLA3 genotype GG (OR = 2.07; 95% CI: 0.97–4.42; p = 0.0599) as the factors independently associated with cirrhosis. When 137 patients without past history of interferon treatment were separately assessed, multivariable logistic regression analysis selected older age (OR = 1.05; 95% CI: 1.02–1.09; p = 0.0034), and PNPLA3 genotype GG (OR = 3.35; 95% CI: 1.13–9.91; p = 0.0291) as the factors independently associated with cirrhosis. Multivariable logistic regression analysis selected older age (OR = 1.12; 95% CI: 1.07–1.17; p < 0.0001), PNPLA3 genotype GG (OR = 2.62; 95% CI: 1.15–5.96; p = 0.0218), and male gender (OR = 1.83; 95% CI: 0.90–3.71); p = 0.0936) as the factors independently associated with HCC.

**Conclusion:**

PNPLA3 genotype I148M is one of risk factors for developing HCC in Japanese CHC patients, and is one of risk factors for progress to cirrhosis in the patients without past history of interferon treatment.

## Background

It is estimated that 130–170 million people, approximately 2%–3% of the world’s population, are infected with the hepatitis C virus (HCV) (Shepard et al. 2005). Of the >500,000 new cases of hepatocellular carcinoma (HCC) that occur each year, approximately 25% are attributable to HCV infection (Block et al. 2003). In Japan, there are an estimated 880,000 HCV carriers aged 16–69 years, and 33,000 deaths occurred each year because of HCC, 81% of which were attributed to HCV infection (Yoshizawa et al. 2006). Treatments for chronic hepatitis C (CHC) have improved and the sustained virological response (SVR) rate has increased to 73%–86% (Fried et al. 2013; Wada et al. 2014). However, HCC still occurs in a large number of HCV carriers. Therefore, the elucidation of factors associated with the development of HCC is still an important task to be continued.

The rs738409 single nucleotide polymorphism (SNP) encoding for a functional I148M protein variant of the patatin-like phospholipase domain-containing 3 (PNPLA3, adiponutrin) gene is associated with hepatic steatosis, inflammation, fibrosis, and carcinogenesis in nonalcoholic fatty liver disease (NAFLD) (Romeo et al. 2008; Rotman et al. 2010; Kawaguchi et al. 2012; Kitamoto et al. 2013). This PNPLA3 gene polymorphism has also been reported to be associated with hepatic steatosis, fibrosis, treatment response, and carcinogenesis with CHC (Valenti et al. 2011; Trepo et al. 2011; Cai et al. 2011; Valenti et al. 2012; Clark et al. 2012; Dunn et al. 2014; Ezzikouri et al. 2014; Moritou et al. 2013; Zampino et al. 2013; Trepo et al. 2014; Sato et al. 2013). However, several reports did not find an association of this PNPLA3 gene polymorphism and some pathological features in CHC (Trepo et al. 2011; Nischalke et al. 2011; Rembeck et al. 2012; Miyashita et al. 2012; Takeuchi et al. 2013; Nakamura et al. 2013; Guyot et al. 2013). Therefore, the association of this PNPLA3 gene polymorphism and pathological features remains to be validated.

Although liver biopsy is the gold standard for diagnosing liver fibrosis, it is an invasive procedure and incurs a high cost. Therefore, it is difficult to perform liver biopsies when numerous patients are involved. However, noninvasive methods have been developed to assess liver fibrosis. Liver stiffness measurements (LSM) by transient elastography (TE) with a Fibroscan (Arima et al. 2010) and the velocity of a shear wave (Vs) measured by an acoustic radiation force impulse (ARFI) (Friedrich-Rust et al. 2009; Nishikawa et al. 2014) are correlated with the liver fibrosis stage in various liver diseases.

In the present study, we investigated possible associations of a PNPLA3 gene polymorphism with fibrosis and the development of HCC in Japanese patients with CHC. We used ARFI to assess hepatic fibrosis.

## Results

### PNPLA3 genotypes

**Figure 1:**
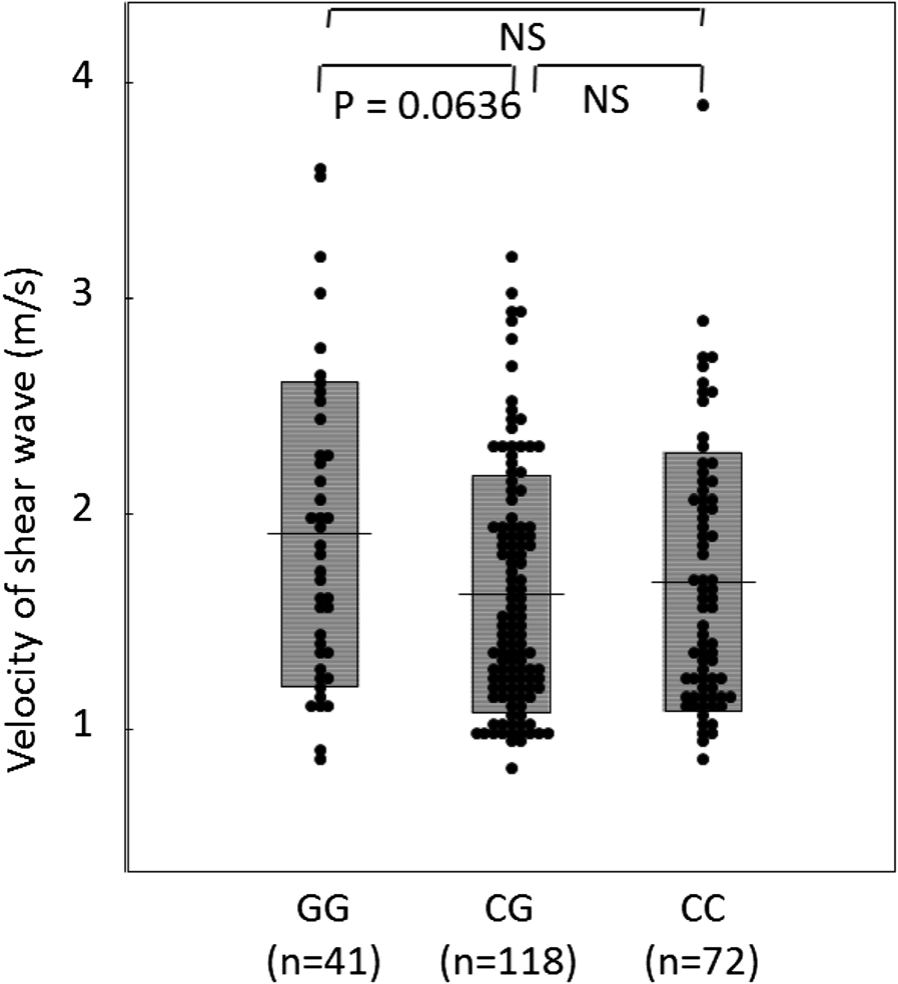
**Vs values among PNPLA3 genotypes.** Vs values tended to be higher for patients with GG than for those with CG (p = 0.0636) and was higher than for those with CC, although there was no statistically significant difference.

**Figure 2:**
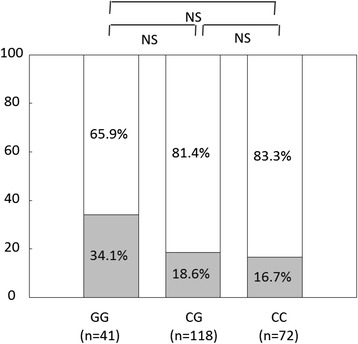
**Frequencies of hepatocellular carcinoma among PNPLA3 genotypes.** The hepatocellular carcinoma frequency was higher for patients with GG than for those with CG or for those with CC, although there was no statistically significant difference.

For PNPLA3 (rs738409 C > G) genotypes, 118 patients had CC, 72 had CG, and 41 had GG. The G allele frequency was 33.3%. Vs values tended to be higher for patients with GG than for those with CG (p = 0.0636) and were higher than for those with CC, although there was no statistically significant difference (Figure 1). The frequency of HCC was higher among patients with GG than among those with CG or CC, although there was no statistically significant difference (Figure 2). Therefore, subsequent comparisons were made between patients with GG and those with CG or CC.

The patients with GG tended to have higher aspartate aminotransferase (AST) levels (p = 0.0946) and higher total bilirubin levels (p = 0.0876) and had significantly lower platelet counts (p = 0.0276), lower prothrombin times (p = 0.0407), higher hyaluronic acid levels (p = 0.0365), higher Vs values (p = 0.0126), and a higher frequency of HCC (p = 0.0200) than those with CG or CC (Table 1).

**Table 1:** **Characteristics of 231 patients studied and comparison among PNPLA3 genotypes**

	**All patients**	**Patients with GG**	**Patients with CC or CG**	**Comparison between patients with GG and those with CG or CG**
	**(n = 231)**	**(n = 41)**	**(n = 190)**	
Age (yrs)	62.9 ± 11.3	63.8 ± 10.6	62.7 ± 11.5	NS
Gender (male/female)	103/128	19/22	84/106	NS
BMI (kg/m^2^)	22.5 ± 3.5	23.3 ± 3.6	22.3 ± 3.5	NS
Response to IFN treatment (NVR/no past IFN therapy)	94/137	19/22	75/115	NS
PNPLA3 (GG/CG/CC)	41/118/72			
AST (IU/L)	57.0 ± 48.8	68.5 ± 61.9	54.5 ± 45.3	p = 0.0946
ALT (IU/L)	62.9 ± 76.1	63.8 ± 51.6	62.7 ± 80.5	NS
γ-GTP (IU/L)	58.9 ± 78.1	60.6 ± 63.5	58.5 ± 81.0	NS
Albumin (g/dL)	4.1 ± 0.6	4.0 ± 0.6	4.1 ± 0.6	NS
Total bilirubin (mg/dL)	1.0 ± 0.8	1.2 ± 0.8	0.9 ± 0.8	p = 0.0876
Platelet count (x10^4^/μL)	13.4 ± 5.6	11.7 ± 5.4	13.8 ± 5.5	p = 0.0276
Prothrombin time (%)	94.3 ± 18.6	88.9 ± 17.7	95.5 ± 18.7	p = 0.0407
Hyaluronic acid (ng/mL)	236.6 ± 337.7	340.5 ± 490.3	214.5 ± 292.3	p = 0.0365
α-fetoprotein (ng/mL)	79.1 ± 529.3	66.3 ± 193.5	81.9 ± 578.0	NS
PIVKA-II(mAU/mL)	24.7 ± 23.1	29.3 ± 36.4	23.7 ± 18.9	NS
HCV genotype (1/2/3)	188/41/2	34/7/0	150/37/2	NS
HCV RNA (log IU/mL)	6.1 ± 1.0	6.1 ± 1.3	6.1 ± 1.0	NS
Velocity of shear wave (m/s)	1.66 ± 0.52	1.91 ± 0.70	1.65 ± 0.57	p = 0.0126
Hepatocellular carcnoma (present/absent)	48/183	14/27	22/96	p = 0.0200

PNPLA3, patatin-like phospholipase domain-containing 3; BMI, body mass index; IFN, interferon; NVR, non-virological response; AST, aspartate aminotransferase; ALT, alanine aminotransferase; γ-GTP, γ-glutamyltranspeptidase; PIVKA-II, protein induced by Vitamin K absence or antagonist-II; Vs, velocity of shear wave; NS, not significant.

### Factors associated with cirrhosis estimated by ARFI

Vs cut-off values for cirrhosis were set at 1.55 m/s, based on a report by Sporea et al.(Sporea et al. 2012). A total of 117 patients had Vs values of ≥ 1.55 m/s and were considered to have liver cirrhosis. As shown in Table 2, cirrhosis was associated with older age (p < 0.0001), higher body mass index (BMI) values (p = 0.0281), PNPLA3 genotype GG (p = 0.0318), higher AST levels (p < 0.0001), higher alanine aminotransferase (ALT) levels (p = 0.0195), lower albumin levels (p < 0.0001), lower platelet counts (p < 0.0001), lower prothrombin times (p < 0.0001), higher hyaluronic acid levels (p < 0.0001), higher α-fetoprotein (AFP) levels (p = 0.0452), higher protein induced by the vitamin K absence or antagonist-II (PIVKA-II) levels (p = 0.0023), HCV genotype 1 (p = 0.0283), and the presence of HCC (p < 0.0001).

**Table 2:** **Comparison between the patients with Vs < 1.55 m/s and those with Vs ≧ 1.55 m/s in all the 231 patients**

	**Patients with Vs ≧ 1.55 m/s**	**Patients with Vs < 1.55 m/s**	**Comparison between patients with Vs < 1.55 m/s and those with Vs ≧ 1.55 m/s**	**Multiple regression analysis for factors associated with ≧ 1.55 m/s**	
	**(n = 117)**	**(n = 114)**		**Odds ratio (95% confidence interval)**	**p**
Age (yrs)	66.1 ± 10.0	60.0 ± 11.6	p < 0.0001	1.06 (1.03 - 1.09)	p < 0.0001
Gender (male/female)	51/66	52/62	NS		NS
BMI (kg/m^2^)	23.0 ± 3.7	22.0 ± 3.2	p = 0.0281	1.12 (1.03 - 1.22)	p = 0.0082
Response to IFN treatment (NVR/no past IFN therapy)	45/72	49/65	NS		
PNPLA3 (GG/CC・CG)	27/90	14/100	p = 0.0318	2.07 (0.97 - 4.42)	p = 0.0599
AST (IU/L)	70.2 ± 47.3	42.4 ± 46.4	p < 0.0001		
ALT (IU/L)	74.0 ± 79.0	50.64 ± 71.3	p = 0.0195		
γ-GTP (IU/L)	61.2 ± 50.2	56.4 ± 100.4	NS		
Albumin (g/dL)	3.8 ± 0.7	4.4 ± 0.4	p < 0.0001		
Total bilirubin (mg/dL)	1.03 ± 0.56	0.87 ± 1.05	NS		
Platelet count (x10^4^/μL)	10.7 ± 4.4	16.3 ± 5.2	p < 0.0001		
Prothrombin time (%)	88.0 ± 14.8	101.2 ± 20.0	p < 0.0001		
Hyaluronic acid (ng/mL)	348.1 ± 330.7	123.0 ± 306.7	p < 0.0001		
α-fetoprotein (ng/mL	146.0 ± 726.0	5.30 ± 5.2	p = 0.0452		
PIVKA-II(mAU/mL)	29.3 ± 29.8	20.0 ± 10.9	p = 0.0023		
HCV genotype (1/2/3)	101/14/2	87/27/0	p = 0.0283		NS
HCV RNA (log IU/mL)	6.2 ± 0.9	6.1 ± 1.1	NS		
Velocity of shear wave (m/s)	1.98 ± 0.46	1.27 ± 0.26	p < 0.0001		
Hepatocellular carcnoma (present/absent)	42/75	6/108	p < 0.0001		

Vs, velocity of shear wave; BMI, body mass index; IFN, interferon; NVR, non-virological response; PNPLA3, patatin-like phospholipase domain-containing 3; AST, aspartate aminotransferase; ALT, alanine aminotransferase; γ-GTP, γ-glutamyltranspeptidase; PIVKA-II, protein induced by Vitamin K absence or antagonist-II ; NS, not significant.

Factors possibly associated with the progression to cirrhosis were assessed by multivariable regression analysis (Table 2). These factors included age, gender, BMI, PNPLA3 genotype, and HCV genotype. AST, ALT, albumin, platelet count, prothrombin time, hyaluronic acid, AFP, and PIVKA-II that were associated with cirrhosis by univariate analyses were excluded because they were apparently the result of cirrhosis but not the causes for the progression to cirrhosis. Because gender was reported to be associated with progression to cirrhosis (Poynard et al. 1997), gender, which was not associated with cirrhosis by univariate analysis, was included among the factors possibly associated with progression to cirrhosis. This analysis showed that older age (OR = 1.06; 95% CI: 1.03–1.09; p < 0.0001), higher BMI values (OR = 1.12; 95% CI: 1.03–1.22; p = 0.0082), and PNPLA3 genotype GG (OR = 2.07; 95% CI: 0.97–4.42; p = 0.0599) were factors independently associated with progression to cirrhosis, although the association with PNPLA3 genotype GG was only a tendency.

One hundred thirty seven patients without past history of interferon (IFN) treatment were separately assessed. Cirrhosis was associated with age (p = 0.0011), PNPLA3 genotype (p = 0.0113), AST levels (p < 0.0001), ALT levels (p = 0.0027), albumin levels (p < 0.0001), total bilirubin levels (p = 0.0078), platelet counts (p < 0.0001), prothrombin times (p = 0.0002), hyaluronic acid levels (p = 0.0006), AFP levels (p = 0.0553), PIVKA-II levels (p = 0.0072), and the presence of HCC (p < 0.0001) (Table 3). Age, gender, and PNPLA3 genotype were assessed for the factors possibly associated with the progression to cirrhosis by multivariable regression analysis (Table 3). This analysis showed that older age (OR = 1.05; 95% CI: 1.02–1.09; p = 0.0034), and PNPLA3 genotype GG (OR = 3.35; 95% CI: 1.13–9.91; p = 0.0291) were factors independently associated with progression to cirrhosis.

**Table 3:** **Comparison between the patients with Vs < 1.55 m/s and those with Vs ≧ 1.55 m/s in the 137 patients without past history of IFN treatment**

	**Patients with Vs ≧ 1.55 m/s**	**Patients with Vs < 1.55 m/s**	**Comparison between patients with Vs < 1.55 m/s and those with Vs ≧ 1.55 m/s**	**Multiple regression analysis for factors associated with ≧ 1.55 m/s**	
	**(n = 72)**	**(n = 65)**		**Odds ratio (95% confidence interval)**	**p**
Age (yrs)	67.3±10.3	60.9±12.0	P = 0.0011	1.05 (1.02 - 1.09)	p = 0.0034
Gender (male/female)	33/39	31/34	NS		NS
BMI (kg/m^2^)	22.6±3.6	21.7±3.4	NS		
PNPLA3 (GG/CC・CG)	17/55	5/60	P = 0.0113	3.35 (1.13 - 9.91)	p = 0.0291
AST (IU/L)	76.7±56.2	37.3±28.4	p < 0.0001		
ALT (IU/L)	82.9±96.2	43.6±41.1	P = 0.0027		
γ-GTP (IU/L)	64.0±57.3	51.9±77.1	NS		
Albumin (g/dL)	3.7±0.6	4.3±0.4	p < 0.0001		
Total bilirubin (mg/dL)	1.1±0.7	0.8±0.6	P = 0.0078		
Platelet count (x10^4^/μL)	10.4±4.5	15.9±5.3	p < 0.0001		
Prothrombin time (%)	87.1±15.4	99.1±21.5	P = 0.0002		
Hyaluronic acid (ng/mL)	385.0±383.0	148.8±371.4	P = 0.0006		
α-fetoprotein (ng/mL)	233.9±939.1	6.8±10.8	P = 0.0553		
PIVKA-II(mAU/mL)	32.9±36.1	20.0±11.9	P = 0.0072		
HCV genotype (1/2/3)	61/10/1	48/17/0	NS		
HCV RNA (log IU/mL)	6.0±0.9	6.2±1.3	NS		
Velocity of shear wave (m/s)	2.14±0.46	1.23±1.0	p < 0.0001		
Hepatocellular carcnoma (present/absent)	28/44	3/62	p < 0.0001		

Vs, velocity of shear wave; BMI, body mass index; IFN, interferon; PNPLA3, patatin-like phospholipase domain-containing 3; AST, aspartate aminotransferase; ALT, alanine aminotransferase; γ-GTP, γ-glutamyltranspeptidase; PIVKA-II, protein induced by Vitamin K absence or antagonist-II ; NS, not significant.

Ninety four patients with non-virological response (NVR) of past IFN treatment were separately assessed. Cirrhosis was associated with age (p = 0.0026), BMI values (p = 0.0274), albumin levels (p < 0.0001), platelet counts (p < 0.0001), prothrombin times (p < 0.0001), hyaluronic acid levels (p < 0.0001), AFP levels (p < 0.0001), and the presence of HCC (p = 0.0017) (Table 4). Age, gender, and BMI were assessed for the factors possibly associated with the progression to cirrhosis by multivariable regression analysis (Table 4). This analysis showed that older age (OR = 1.08; 95% CI: 1.03–1.13; p = 0.0023), and higher BMI values (OR = 1.20; 95% CI: 1.04–1.39; p = 0.0156) were factors independently associated with progression to cirrhosis.

**Table 4:** **Comparison between the patients with Vs < 1.55 m/s and those with Vs ≧ 1.55 m/s in the 94 patients with NVR of past IFN treatment**

	**Patients with Vs ≧ 1.55 m/s**	**Patients with Vs < 1.55 m/s**	**Comparison between patients with Vs < 1.55 m/s and those with Vs ≧ 1.55 m/s**	**Multiple regression analysis for factors associated with ≧ 1.55 m/s**	
	**(n = 45)**	**(n = 49)**		**Odds ratio (95% confidence interval)**	**p**
Age (yrs)	64.3±9.4	57.7±10.9	P = 0.0026	1.03 (1.03 - 1.13)	p = 0.0023
Gender (male/female)	18/27	21/28	NS		NS
BMI (kg/m^2^)	23.7±3.8	22.2±2.8	P = 0.0274	1.20 (1.04 - 1.39)	p = 0.0156
PNPLA3 (GG/CC・CG)	10/35	9/40	NS		
AST (IU/L)	59.6±22.0	51.7±63.9	NS		
ALT (IU/L)	58.6±31.2	62.8±98.8	NS		
γ-GTP (IU/L)	57.2±37.1	62.1±122.7	NS		
Albumin (g/dL)	3.9±0.7	4.4±0.3	p < 0.0001		
Total bilirubin (mg/dL)	0.9±0.3	1.0±1.4	NS		
Platelet count (x10^4^/μL)	11.1±4.4	16.7±5.1	p < 0.0001		
Prothrombin time (%)	88.5±14.1	104.0±16.5	p < 0.0001		
Hyaluronic acid (ng/mL)	305.2±229.7	89.3±170.5	p < 0.0001		
α-fetoprotein (ng/mL)	20.1±18.0	4.9±3.6	p < 0.0001		
PIVKA-II(mAU/mL)	23.5±13.9	19.9±9.3	NS		
HCV genotype (1/2/3)	40/4/1	39/10/0	NS		
HCV RNA (log IU/mL)	6.3±0.9	6.0±1.3	NS		
Velocity of shear wave (m/s)	2.20±0.50	1.23±0.16	p < 0.0001		
Hepatocellular carcnoma (present/absent)	14/31	3/46	P = 0.0017		

NVR, non-virological response; IFN, interferon; Vs, velocity of shear wave; BMI, body mass index; PNPLA3, patatin-like phospholipase domain-containing 3; AST, aspartate aminotransferase; ALT, alanine aminotransferase; γ-GTP, γ-glutamyltranspeptidase; PIVKA-II, protein induced by Vitamin K absence or antagonist-II ; NS, not significant.

### Factors associated with the development of HCC

As shown in Table 5, HCC was associated with older age (p < 0.0001), PNPLA3 genotype GG (p = 0.0200), higher AST levels (p = 0.0158), lower albumin levels (p < 0.0001), higher total bilirubin levels (p = 0.0010), lower platelet counts (p < 0.0001), lower prothrombin times (p < 0.0001), higher hyaluronic acid levels (p < 0.0001), higher AFP levels (p = 0.0009), higher PIVKA-II levels (p = 0.0030), and higher Vs values (p < 0.0001).

**Table 5:** **Comparison between the patients with HCC and those without HCC in all the 231 patients**

	**Patients with HCC**	**Patients without HCC**	**Comparison between patients with HCC and those without HCC**	**Multiple regression analysis for factors associated with HCC development**	
	**(n = 48)**	**(n = 183)**		**Odds ratio (95% confidence interval)**	**p**
Age (yrs)	70.5 ± 7.8	60.9 ± 11.2	p < 0.0001	1.12 (1.07 - 1.17)	p < 0.0001
Gender (male/female)	25/23	78/105	NS	1.83 (0.90 - 3.71)	p = 0.0936
BMI (kg/m^2^)	23.1 ± 3.9	22.3 ± 3.4	NS		
Response to IFN treatment (NVR/no past IFN therapy)	17/31	77/106	NS		
PNPLA3 (GG/CC・CG)	34/14	156/27	p = 0.0200	2.62 (1.15 - 5.96)	p = 0.0218
AST (IU/L)	72.1 ± 54.8	53.0 ± 46.4	p = 0.0158		
ALT (IU/L)	60.4 ± 46.4	63.5 ± 82.1	NS		
γ-GTP (IU/L)	50.4 ± 34.9	61.1 ± 85.8	NS		
Albumin (g/dL)	3.5 ± 0.7	4.2 ± 0.5	p < 0.0001		
Total bilirubin (mg/dL)	1.3 ± 0.97	0.9 ± 0.76	p = 0.0010		
Platelet count (x10^4^/μL)	9.5 ± 4.2	14.4 ± 5.4	p < 0.0001		
Prothrombin time (%)	83.7 ± 13.90	97.1 ± 18.8	p < 0.0001		
Hyaluronic acid (ng/mL)	473.8 ± 480.9	181.1 ± 267.2	p < 0.0001		
α-fetoprotein (ng/mL)	308.6 ± 1134.9	20.8 ± 113.2	p = 0.0009		
PIVKA-II(mAU/mL)	40.25 ± 43.22	20.56 ± 9.93	p = 0.0030		
HCV genotype (1/2/3)	42/6/0	169/35/2	NS		
HCV RNA (log IU/mL)	6.0 ± 1.1	6.2 ± 1.0	NS		
Velocity of shear wave (m/s)	2.19 ± 0.64	1.57 ± 0.52	p < 0.0001		

HCC, hepatocellular carcinoma; BMI, body mass index; IFN, interferon; NVR, non-virological response; PNPLA3, patatin-like phospholipase domain-containing 3; AST, aspartate aminotransferase; ALT, alanine aminotransferase; γ-GTP, γ-glutamyltranspeptidase; PIVKA-II, protein induced by Vitamin K absence or antagonist-II; NS, not significant.

Factors possibly associated with the development of HCC were assessed by multivariable regression analysis (Table 5). These factors included age, gender, and PNPLA3 genotype. AST, albumin, total bilirubin, platelet count, prothrombin time, hyaluronic acid, AFP, PIVKA-II, and Vs that were associated with HCC by univariate analyses were excluded because they were apparently the result of cirrhosis, which is a major risk factor for HCC, but not the causes of cirrhosis or development of HCC. Because gender was reported to be associated with the development of HCC (Asahina et al. 2010), gender, which was not associated with HCC by univariate analysis, was included for multivariable analysis. This analysis showed that older age (OR = 1.12; 95% CI: 1.07–1.17; p < 0.0001), PNPLA3 genotype GG (OR = 2.62; 95% CI: 1.15–5.96; p = 0.0218), and male gender (OR = 1.83; 95% CI: 0.90–3.71; p = 0.0936) were factors independently associated with the development of HCC, although the association with gender was only a tendency.

One hundred thirty seven patients without past history of IFN treatment were separately assessed. HCC was associated with age (p = 0.0002), PNPLA3 genotype (p = 0.0928), AST levels (p = 0.0032), albumin levels (p < 0.0001), total bilirubin levels (p < 0.0001), platelet counts (p = 0.0008), prothrombin times (p = 0.0002), hyaluronic acid levels (p = 0.0002), AFP levels (p = 0.0016), PIVKA-II levels (p < 0.0001), and Vs values (p < 0.0001) (Table 6). Age, gender, and PNPLA3 genotype were assessed for factors possibly associated with the development of HCC by multivariable regression analysis (Table 6). This analysis showed that older age (OR = 1.09; 95% CI: 1.04–1.15; p = 0.0006) was an only factor independently associated with the development of HCC.

**Table 6:** **Comparison between the patients with HCC and those without HCC in the 137 patients without past history of IFN treatment**

	**Patients with HCC**	**Patients without HCC**	**Comparison between patients with HCC and those without HCC**	**Multiple regression analysis for factors associated with HCC development**	
	**(n = 31)**	**(n = 106)**		**Odds ratio (95% confidence interval)**	**p**
Age (yrs)	70.9±7.1	62.3±11.9	P = 0.0002	1.09 (1.04 - 1.15)	p = 0.0006
Gender (male/female)	16/15	48/58	NS		NS
BMI (kg/m^2^)	23.1±4.0	21.9±3.4	NS		
PNPLA3 (GG/CC・CG)	8/23	14/92	P = 0.0928		NS
AST (IU/L)	80.7±64.3	51.4±41.8	P = 0.0032		
ALT (IU/L)	65.5±50.0	63.9±84.1	NS		
γ-GTP (IU/L)	53.5±40.7	60.0±73.6	NS		
Albumin (g/dL)	3.4±0.56	4.2±0.5	p < 0.0001		
Total bilirubin (mg/dL)	1.5±1.1	0.8±0.4	p < 0.0001		
Platelet count (x10^4^/μL)	9.2±4.3	14.1±5.5	P = 0.0008		
Prothrombin time (%)	81.5±14.0	96.0±19.7	P= 0.0002		
Hyaluronic acid (ng/mL)	507.0±542.5	202.1±313.7	P = 0.0002		
α-fetoprotein (ng/mL)	475.9±1411.2	28.7±148.1	P = 0.0016		
PIVKA-II(mAU/mL)	47.0±51.0	20.8±10.7	p < 0.0001		
HCV genotype (1/2/3)	27/4/0	82/23/1	NS		
HCV RNA (log IU/mL)	5.9±1.0	6.2±0.9	NS		
Velocity of shear wave (m/s)	2.20±0.70	1.55±0.50	p < 0.0001		

HCC, hepatocellular carcinoma; IFN, interferon; BMI, body mass index; NVR, non-virological response; PNPLA3, patatin-like phospholipase domain-containing 3; AST, aspartate aminotransferase; ALT, alanine aminotransferase; γ-GTP, γ-glutamyltranspeptidase; PIVKA-II, protein induced by Vitamin K absence or antagonist-II; NS, not significant.

Ninety four patients with NVR of past IFN treatment were separately assessed. HCC was associated with age (p < 0.0001), PNPLA3 genotype (p = 0.0871), albumin levels (p < 0.0001), platelet counts (p = 0.0008), prothrombin times (p = 0.0295), hyaluronic acid levels (p = 0.0002), AFP levels (p = 0.0005), PIVKA-II levels (p = 0.0134), and Vs values (p = 0.0002) (Table 7). Age, gender, and PNPLA3 genotype were assessed for the factors possibly associated with the development of HCC by multivariable regression analysis (Table 7). This analysis showed that older age (OR = 1.19; 95% CI: 1.08–1.32; p = 0.0007), and PNPLA3 genotype GG (OR = 3.95; 95% CI: 1.00–15.61; p = 0.0497) were factors independently associated with the development of HCC.

**Table 7:** **Comparison between the patients with HCC and those without HCC in the 94 patients with NVR of past IFN treatment**

	**Patients with HCC**	**Patients without HCC**	**Comparison between patients with HCC and those without HCC**	**Multiple regression analysis for factors associated with HCC development**	
	**(n = 17)**	**(n = 77)**		**Odds ratio (95% confidence interval)**	**p**
Age (yrs)	69.6±9.3	58.9±10.0	P = 0.0001	1.19 (1.08 - 1.32)	p = 0.0007
Gender (male/female)	9/8	47/30	NS		NS
BMI (kg/m^2^)	23.2±3.8	22.9±3.3	NS		
PNPLA3 (GG/CC・CG)	6/11	13/64	P = 0.0871	3.95 (1.00 - 15.61)	p = 0.0497
AST (IU/L)	56.35±25.5	55.3±52.3	NS		
ALT (IU/L)	51.1±38.6	63.0±80.0	NS		
γ-GTP (IU/L)	44.8±20.6	63.1±100.8	NS		
Albumin (g/dL)	3.5±1.0	4.3±0.4	p < 0.0001		
Total bilirubin (mg/dL)	0.9±0.5	0.9±1.1	NS		
Platelet count (x10^4^/μL)	10.1±4.1	14.9±5.4	P = 0.0008		
Prothrombin time (%)	88.0±14.2	98.4±17.3	P = 0.0295		
Hyaluronic acid (ng/mL)	402.2±316.9	153.1±186.6	P = 0.0002		
α-fetoprotein (ng/mL)	23.2±20.6	9.8±12.1	P = 0.0005		
PIVKA-II(mAU/mL)	28.0±19.4	20.2±8.9	P = 0.0134		
HCV genotype (1/2/3)	16/1/0	63/13/1	NS		
HCV RNA (log IU/mL)	6.2±1.2	6.2±1.1	NS		
Velocity of shear wave (m/s)	2.20±0.70	1.60±0.5	P = 0.0002		

HCC, hepatocellular carcinoma; IFN, interferon; NVR, non-virological response; BMI, body mass index; PNPLA3, patatin-like phospholipase domain-containing 3; AST, aspartate aminotransferase; ALT, alanine aminotransferase; γ-GTP, γ-glutamyltranspeptidase; PIVKA-II, protein induced by Vitamin K absence or antagonist-II; NS, not significant.

## Discussion

In this study, we demonstrated that a PNPLA3 gene polymorphism was associated with the progression of fibrosis to cirrhosis and development of HCC, although the association with cirrhosis was only a tendency by multivariable analysis in all the 231 patients studied. When the patients without past IFN treatment and those with NVR of past IFN treatment were separately analyzed, a PNPLA3 gene polymorphism was selected as a factor independently associated with progression to cirrhosis in those without previous IFN treatment but not in those with NVR of past IFN treatment. A PNPLA3 gene polymorphism was selected as a factor independently associated with development to HCC in those with NVR of past IFN treatment but not in those without past IFN treatment.

PNPLA3 polymorphisms have been reported to be associated with hepatic steatosis, inflammation, fibrosis, and carcinogenesis in NAFLD (Romeo et al. 2008; Rotman et al. 2010; Valenti et al. 2010; Sookoian and Pirola 2011; Burza et al. 2012; Kawaguchi et al. 2012; Kitamoto et al. 2013). PNPLA3 polymorphisms have also been reported to be associated with hepatic steatosis, fibrosis, treatment response, and carcinogenesis in CHC (Valenti et al. 2011; Trepo et al. 2011; Cai et al. 2011; Valenti et al. 2012; Clark et al. 2012; Dunn et al. 2014; Ezzikouri et al. 2014; Moritou et al. 2013; Zampino et al. 2013; Trepo et al. 2014; Sato et al. 2013). However, several reports have not found an association of PNPLA3 polymorphisms with fibrosis and carcinogenesis in CHC (Trepo et al. 2011; Nischalke et al. 2011; Rembeck et al. 2012; Miyashita et al. 2012; Takeuchi et al. 2013; Nakamura et al. 2013; Guyot et al. 2013).

A significant association was reported between a PNPLA3 polymorphism and HCC in patients with CHC (Valenti et al. 2011; Ezzikouri et al. 2014), while other studies did not find a significant association(Nischalke et al. 2011; Guyot et al. 2013). A meta-analysis performed by Trepo et al. showed that a PNPLA3 polymorphism was strongly associated with HCC, although the association was stronger in patients with alcoholic liver disease (OR = 2.20; 95% CI: 1.802.67; P = 4.71 × 10^−15^) than that in patients with CHC (OR = 1.55; 95% CI: 1.03–2.34; P = 3.52 × 10^−2^) (Trepo et al. 2014). In Japanese studies, Moritou et al. reported that a PNPLA3 polymorphism was significantly associated with serum AFP level (Moritou et al. 2013), and Sato et al. reported that the median time between HCV infection and the development of HCC was significantly shorter for patients with the PNPLA3 GG genotype in HCV-related HCC (Sato et al. 2013). The results of our present study confirmed an association between PNPLA3 and the development of HCC in Japanese patients.

In this study, we demonstrated that a PNPLA3 polymorphism was associated with the progression of fibrosis to cirrhosis. The association was significant by multivariable analysis in the patients without past IFN treatment, while it was only a tendency by analysis in all the 231 patients studied.

Several studies reported that a PNPLA3 polymorphism was associated with fibrosis in patients with CHC (Valenti et al. 2011; Trepo et al. 2011; Valenti et al. 2012; Dunn et al. 2014), while other studies did not find an association between a PNPLA3 polymorphism and fibrosis (Zampino et al. 2013; Rembeck et al. 2012; Miyashita et al. 2012; Nakamura et al. 2013).

These discrepancies reported on the association of PNPLA3 with the development of HCC or fibrosis may be attributed to the difference of the ethnicity, population, and past treatment of the patients studied. In our study, the patients with SVR and relapse of past IFN treatment were excluded, because their LSM results declined and the risk of the development of HCC also was reduced (Arima et al. 2010; Kasahara et al. 1998; Harada et al. 2014). Because the associations of PNPLA3 are not strong for fibrosis (OR = 3.13; 95% CI: 1.50–6.51; P = 0.002) (Trepo et al. 2011) and development of HCC (Trepo et al. 2014), a large number of more homogenous patients should be studied to establish an association by statistical analysis. The present study included 231 patients, and the association between fibrosis and PNPLA3 was shown to be only a tendency by multivariable analysis, while the association was shown by multivariable analysis of the patients without past IFN treatment.

In our present study, we diagnosed cirrhosis on the basis of Vs values rather than by liver biopsy. Some studies reported that an association between Vs values and fibrosis is affected by inflammation (Chen et al. 2012; Yoon et al. 2012), although others denied this association (Bota et al. 2013; Nishikawa et al. 2014; Rizzo et al. 2011). To confirm this association between fibrosis and PNPLA3 in Japanese patients, further studies using liver biopsies are required.

Nishikawa et al. reported that Vs values were negatively correlated with BMI in the patients with fibrosis stage F1 or F2, but not in those with F3 or F4 (Nishikawa et al. 2014). Bota et al. reported that higher BMI (≥27.7 kg/m^2^) were associated with the risk of failed and unreliable measurements of ARFI (Bota et al. 2014). In the present study, BMI was ≥ 27.7 kg/m^2^ in 17 patients. Thus we analyzed the 214 patients with BMI < 27.7 kg/m^2^. Multivariate analysis showed that older age (OR = 1.06; 95% CI: 1.03–1.09; p = 0.0001), higher BMI (OR = 1.11; 95% CI: 1.00–1.24; p = 0.0576), and PNPLA3 genotype GG (OR = 2.07; 95% CI: 0.94–4.55; p = 0.0712) were factors independently associated with progression to cirrhosis (data not shown). The standard range of BMI is 18.5 – 24.9 kg/m^2^. Thus we analyzed 154 patients with BMI of 18.5 – 24.9 kg/m^2^. Neither univariate nor multivariate analysis showed the association of PNPLA3 genotype with cirrhosis (data not shown).

The mechanism underlying the association between a PNPLA3 gene polymorphism with the progression of steatosis, fibrosis, and development of HCC has not been determined. It was recently reported that a PNPLA3 I148M variant promotes the synthesis of hepatic lipid because of a gain of function (Kumari et al. 2012). Steatosis maintained by the PNPLA3 genotype I148M may promote the progression of fibrosis and development of HCC (Valenti et al. 2011; Trepo et al. 2011; Valenti et al. 2012).

## Conclusions

In this study, we confirmed that the PNPLA3 genotype I148M was associated with the development of HCC in Japanese patients with CHC, and is one of risk factors for cirrhosis in the patients without past history of IFN treatment. Further studies are required to clarify the mechanism underlying this association.

## Methods

### Patients

Two hundred thirty-one patients with chronic HCV infection consulted with the Department of Liver, Biliary Tract and Pancreas Diseases, Fujita Health University Hospital from May 2010 to October 2012 (Table 1). Of these patients, 137 had no past history of IFN treatment. The other 94 patients had a past history of IFN treatment, for which HCV RNA did not become negative during treatment and their results were considered as NVR. The patients with a past history of IFN treatment and who had achieved a SVR or relapse, which indicated temporary HCV RNA negativity during the treatment, were excluded from the present study because their LSM results declined and the risk of the development of HCC also reduced (Arima et al. 2010; Kasahara et al. 1998; Harada et al. 2014).

In addition, patients with hepatitis B virus coinfection, human immunodeficiency virus coinfection, alcoholic liver disease, or autoimmune liver disease were not included in the study. This study was approved by the ethics committee of the Fujita Health University and was conducted in accordance with the Declaration of Helsinki of 1975, as revised in 2008. All patients who participated in this study had provided written informed consent.

### PNPLA3 rs738409 genotyping

Genomic DNA was extracted from whole blood samples using QIA amp DNA Mini Kits (Qiagen, Tokyo, Japan), according to the manufacturer’s protocol. The rs738409 PNPLA3 SNP was genotyped using TaqMan predesigned SNP genotyping assays (Applied Biosystems, Tokyo, Japan), according to the manufacturer’s protocol.

### ARFI measurements

Vs measurements by ARFI were made with a Siemens ACUSON S2000 (Siemens Japan Co., Ltd., Tokyo, Japan) as previously reported (Nishikawa et al. 2014). Vs values were expressed in meters/second (m/s), and was considered to be proportional to the square root of tissue elasticity.

### Statistical analysis

Results are expressed as means ± standard deviations. Group results were compared using chi-square test or Student’s *t*-test, as appropriate. Bonferroni corrections were used during multiple group comparisons. Factors possibly associated with Vs of ≥1.55 m/s or with the development of HCC were assessed using stepwise logistic regression analysis. Statistical analysis was performed using the StatFlex version 5.0 for Windows (StatFlex, Osaka Japan). A two-sided p-value of <0.05 was considered significant.

## Abbreviations

PNPLA3: Patatin-like phospholipase domain-containing 3
CHC: Chronic hepatitis C
LSM: Liver stiffness measurements
HCC: Hepatocellular carcinoma
SNP: Single nucleotide polymorphism
Vs: Velocity of a shear wave
BMI: Body mass index
IFN: Interferon
HCV: Hepatitis C virus
SVR: Sustained virological response
NAFLD: Nonalcoholic fatty liver disease
TE: Transient elastography
ARFI: Acoustic radiation force impulse
AST: Aspartate aminotransferase
ALT: Alanine aminotransferase
AFP: α-fetoprotein
PIVKA-II: Vitamin K absence or antagonist-II
NVR: Non-virological response
